# IL12-Mediated Liver Inflammation Reduces the Formation of AAV Transcriptionally Active Forms but Has No Effect over Preexisting AAV Transgene Expression

**DOI:** 10.1371/journal.pone.0067748

**Published:** 2013-07-02

**Authors:** Irene Gil-Fariña, Marianna Di Scala, Lucia Vanrell, Cristina Olagüe, Africa Vales, Katherine A. High, Jesus Prieto, Federico Mingozzi, Gloria Gonzalez-Aseguinolaza

**Affiliations:** 1 Division of Hepatology and Gene Therapy, Center for Applied Medical Research, Pamplona, Spain; 2 Center for Cellular and Molecular Therapeutics at The Children's Hospital of Philadelphia, Howard Hughes Medical Institute, Philadelphia, Pennsylvania, United States of America; 3 Liver Unit and Centro de Investigación Biomédica en Red en el Área temática de Enfermedades Hepáticas y Digestivas, University Clinic of Navarra, La Universidad de Navarra, Pamplona, Spain; Justus-Liebig-University Giessen, Germany

## Abstract

Recombinant adenoassociated viral vectors (rAAV) have proven to be excellent candidates for gene therapy clinical applications. Recent results showed that cellular immunity to AAV represents a major challenge facing the clinical use of systemic administration of these vectors. Interestingly, no preclinical animal model has previously fully reproduced the clinical findings. The aim of the present work was to enhance the T cell immune response against AAV capsid in mice by the administration of a rAAV expressing the immunostimulatory cytokine IL-12. Our results indicate that although IL-12 expression enhanced the AAV capsid-specific immune response it failed to eliminate transduced hepatocytes and long-term expression was achieved. We found that AAV-mediated transgene expression is altered by IL-12-induced liver inflammation. However, IL-12 expression has no effect over preexisting AAV-mediated transgene expression. IL-12 down-regulates AAV mediated transgene expression via induction of IFN-γ production by NK and T cells, but without altering the transduction efficiency measured by viral genomes. Our results indicate that liver inflammation affects the formation of transcriptionally active AAV vector genomes through an unknown mechanism that can be avoided by the use of DNA-demethylating or anti-inflammatory agents.

## Introduction

AAV is a parvovirus, which is a family of small, non-enveloped viruses containing a single-stranded linear DNA genome of about 5 kb; the wild-type virus is replication-deficient, requiring a helper virus for multiplication. In humans, AAV has not been found to be pathogenic. This fact, along with the tendency for the genomes of recombinant AAV (rAAV) vectors to remain as episomal concatemers rather than integrating into the host genome, makes AAV a relatively safe gene transfer vector for testing in the clinic [1]. AAV have been used successfully for in vivo gene transfer in numerous preclinical animal models of human disease. Recently, AAV vectors have also generated long-term clinical benefit in patients suffering from Leber’s congenital amaurosis [2]. However, in patients with hemophilia B, hepatic gene transfer with AAV2 and AAV8 expressing Factor IX induced specific cellular immune responses against the viral capsid that in the case of the AAV2, resulted in loss of expression of Factor IX. In the case of AAV8, the administration of a short course of high dose steroids blocked the immune response and allowed clinical efficacy [3] [4]. None of the preclinical animal models used to test these vectors predicted the outcome in humans, and the low immunogenicity of AAV vectors has been thought to be one of main features of AAV vectors. For example, activation of innate immunity following AAV vector administration is very low and transient [5], although significant differences have been reported depending on the target organ. AAV intramuscular injection induces signaling through the Toll-like receptor 9 (TLR9)–myeloid differentiation factor 88 (MyD88) pathway to induce type I IFN and this activation is critical for CD8+ T cell responses to the AAV capsid and for the loss of transgene expression in vivo [6]. However, in the liver although AAV vectors have been shown to induce the expression of chemokines and cytokines, the response is transient and occurs at a higher threshold titers compared to adenovirus vectors [7]. It is well known that innate immunity plays an important role in the subsequent T cell responses against viruses, since it provides activation signals that recruit and activate antigen presenting cells as well as T and B cell immune responses. IL-12 has the capacity to activate both innate and adaptive immune response [8]. IL-12 has been used as an adjuvant for vaccination and for the treatment of tumors, leading to the generation of specific CTLs [9]. With the aim of inducing a T cell response against the AAV capsid in mice, in the present study we have constructed a recombinant AAV expressing the cytokine IL-12 in the liver, constitutively or after tetracycline induction [9]. Mice were treated with IL-12 expression vectors alone or together with an AAV vector expressing luciferase as reporter gene. Although AAV specific T cell responses could be induced by the expression of IL-12, long-term transgene expression was nonetheless achieved, indicating that the T cell response failed to eliminate transgene expression. Furthermore, we found that AAV-mediated expression is altered by IL-12-induced liver inflammation; in particular, the IFN-γ produced by NK and T cells alters the formation of AAV transcriptionally active forms but has no effect on previously established transgene expression. This effect can be reversed by the administration of DNA-demethylating and anti-inflammatory agents.

## Results

### AAV-mediated Sustained Liver Specific IL-12 Expression

**Figure 1:**
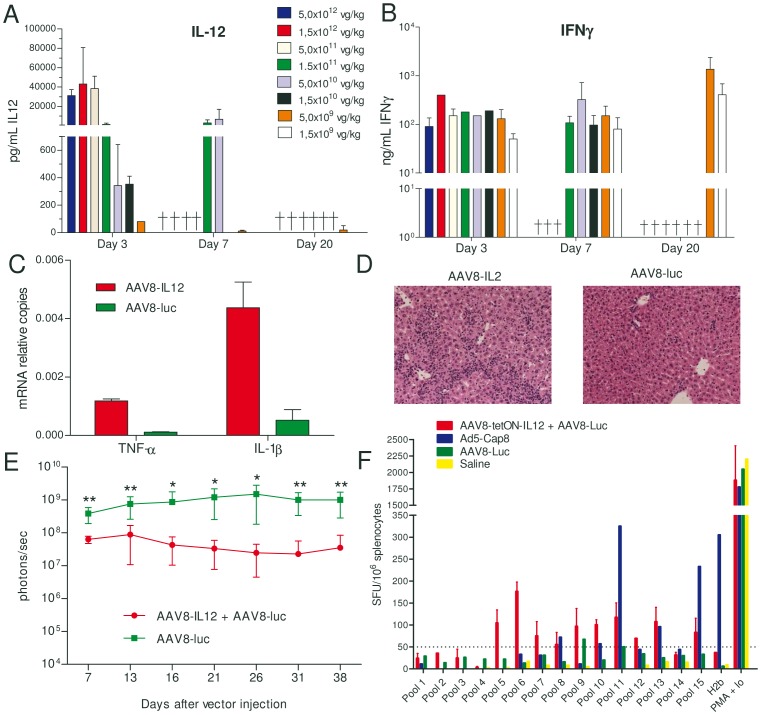
AAV8-IL-12 induces liver inflammation, enhances immune response against AAV8 but do not eliminate transduced hepatocytes. C57BL/6 female mice (n = 5) received intravenously (IV) different doses of AAV8-IL12 vector and IL-12 A) and IFN-γ B) concentration in serum was analyzed by ELISA at different time points after vector injection. C) C57BL/6 female were IV injected with AAV8-IL-12 or AAV8-luc vectors at a dose of 1.5**×**109 vg/kg, 21 days after injection TNFα and IL-1β expression levels were analyzed in the liver by RT qPCR. D) Hematoxilin and eosin staining of liver sections. E) Animals were injected with an AAV8-luc vector at a dose of 2.5×1011 vg/kg with or without coadministration of an AAV8-IL12 vector at a dose of 1.5**×**109 vg/kg. Luciferase expression in the liver was followed for 38 days by in vivo bioluminescence imaging and quantification is represented as photons/second. F) C57BL/6 female mice received the AAV8-tetON-IL12 vector together with an AAV8-luc vector, or AAV8-luc vector alone, or an adenoviral vector expressing AAV8 capsid protein (Ad5-Cap8) or saline. Twenty days after vector injection, animals receiving AAV8-tetON-IL12 and AAV8-luc or AAV8-luc alone were treated with doxycycline (Dox) to induce IL-12 for 7 days expression and the immune response was analyzed 14 days after Tet removal. Animals receiving Ad5-Cap8 were sacrificed 10 days after vector injection. Splenocytes obtained from these mice were stimulated with different pools of peptides derived from the AAV8 VP1 capsid protein, the H2b peptide previously described for the AAV8 capsid protein, NSLANPGIA [10], or PMA plus ionomycin as positive control.

C57BL/6 female mice (n = 5) were intravenously injected with different doses of an AAV8 expressing IL12 under the control of a liver specific promoter, AAV8-IL12, 1.5**×**10^9^, 5**×**10^9^, 1.5**×**10^10^, 5**×**10^10^, 1.5**×**10^11^, 5**×**10^11^, 1.5**×**10^12^, or 5**×**10^12^ viral genomes (vg)/kg. Mice receiving the higher doses died between day three and seven after vector administration due to IL-12 toxicity, characterized by a significant loss of weight and diarrhea. Only the mice receiving the lowest doses, 5**×**10^9^, and 1.5**×**10^9 ^vg/kg, survived with IL-12 serum concentrations close to the ELISA detection limit or undetectable (Fig. 1A). However, IFN-**γ** expression, the main mediator of IL12 activity, could be detected in serum (Fig. 1B). Twenty-one days after AAV8-IL12 injection at a dose of 1.5**×**10^9 ^vg/kg animals were sacrificed and the degree of inflammatory response in the liver was analyzed. High expression levels of TNF-α and IL-1β were detected in the liver (Fig. 1C) together with significant inflammatory cellular infiltrates (Fig. 1D).

### IL-12 Expression Enhances the T–cell Response Against the Viral Capsid but does not Result in Elimination of AAV Mediated Transgene Expression

In order to determine if AAV8-IL12 can induce a T cell specific immune response able to eliminate AAV8 transduced cells, we coadministered the AAV8-IL12 together with an AAV8 vector expressing luciferase (AAV8-luc) that allowed us to monitor long-term transgene expression *in vivo*. C57BL/6 mice were injected with AAV8-luc at a dose of 5**×**10^11^ vg/kg and AAV8-IL12 at a dose of 1.5**×**10^9 ^vg/kg or with AAV8-luc alone (5**×**10^11^ vg/kg). As shown in Fig. 1E, sustained luciferase expression could be detected in both groups of animals. The immune response against peptides derived from the AAV8 capsid protein were analyzed in these animals, however, due to the sustained IL-12 expression, no differences in IFN-**γ** production were observed between non-stimulated or peptide-stimulated lymphocytes due to the sustained expression of IFN-**γ** induced by IL-12. To eliminate the background, an AAV8 expressing IL-12 upon tetracycline administration was used (AAV8-tetON-IL12). Mice received an intravenous injection of AAV8-tetON-IL12 twenty days later IL-12 expression was induced by tetracycline administration during seven days. The immune response was analyzed two weeks after tetracycline removal, once the IFN-**γ** disappeared from serum. As a positive control mice were immunized with an adenoviral vector expressing AAV8 capsid protein (Ad5-Cap8) and 10 days later the animals were sacrificed [10]. ELISpot analysis revealed that inducible IL-12 expression increased T cell response against peptides derived from the AAV8 capsid protein (Fig. 1F). The response against a previously described H2b epitope [10] induced by AAV8-tetON-IL12/AAV8-luc is lower in comparison to the one induced by Ad5-Cap8. However, the total number of IFN-**γ** producing cells after stimulation with the peptide pools covering the whole capsid protein is very similar (Fig. 1F). Interestingly, the pools of peptides that induce IFN-**γ** production in mice treated with AAV8-tetON-IL12+AAV8-luc are different from pools recognized by T cells induced by the adenovirus expressing the AAV8 capsid VP1 protein, suggesting that antigen presentation is different when the capsid protein is expressed in a target cell or when it is endocytosed by antigen presenting cells (Fig. 1F).

### IL-12 Expression Significantly Downregulates AAV Mediated Transgene Expression

**Figure 2:**
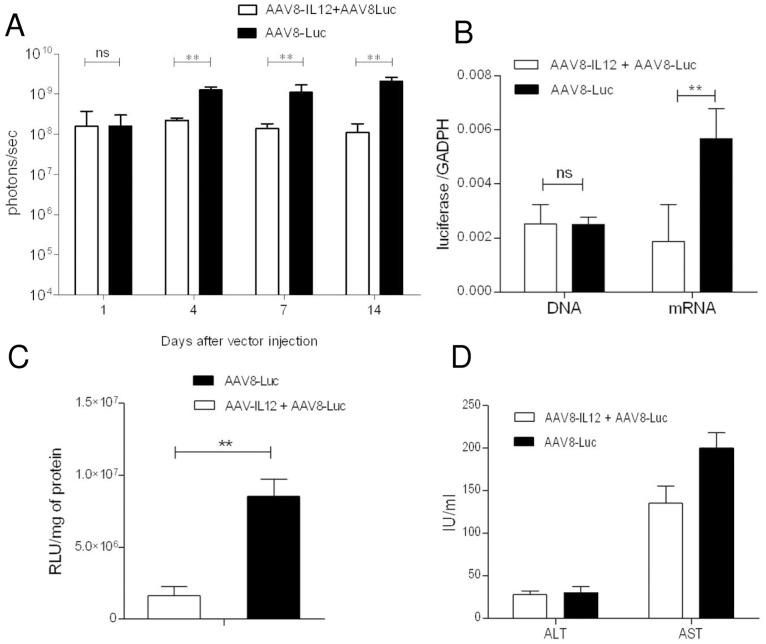
IL-12 down-regulates AAV mediated gene expression without reducing the number of viral genomes. C57BL/6 mice (n = 5) received 2.5**×**1011 vg/kg of AAV8-luc vector alone or in combination with 1.5**×**109 vg/kg of AAV8-IL12 vector. A) Luciferase expression levels were analyzed in both groups of animals at days 1, 4, 7, and 14 using a Xenogen in vivo luminometer. Luciferase expression was quantified and represented as photons/second. The data are shown as mean ± standard deviation (SD). The differences in luciferase expression were statistically evaluated by Student t test (**p<0.01). B) Animals were sacrificed at day 14. Total DNA and RNA were isolated from liver and vector genome copies and luciferase messenger RNA (mRNA) copies were determined using a q-PCR specific for luciferase, normalized to GADPH copy numbers. Values correspond to the mean copy number from five animals. C) The levels of luciferase activity (relative light units, RLUs) were determined in liver homogenates and were normalized to protein content (RLU/mg). The data are shown as mean ± SEM. D) Serum transaminases were analyzed 14 days after vector injection. The data are shown as mean ± standard deviation (SD). Each experiment was repeated three times.

As shown in Fig. 1E, IL-12 expression does not eliminate but significantly downregulates luciferase expression and the effect is maintained over time. To further characterize the effect of IL-12 on transgene expression C57BL/6 mice were injected with the IL12- and the luciferase-encoding vectors as previously described, and luciferase expression was analyzed at 1, 4, 7, and 14 days after viral injection. No significant differences were observed at day 1 between the two groups (Fig. 2A). At day 4 the group of animals that received both vectors showed a significantly lower level of luciferase expression than the animals receiving luciferase alone. The differences between the two groups increased with time (Fig. 2A), and at day 14 luciferase expression values were tenfold lower in the group of animals receiving IL-12 and luciferase compared to the animals receiving luciferase alone.

Mice were sacrificed at day 14 and viral genome and luciferase mRNA expression levels were analyzed by quantitative PCR (qPCR), and luciferase activity was determined in liver extracts. As shown in Fig. 2B, the number of viral genomes was the same in both groups, while luciferase mRNA levels (Fig. 2B) and luciferase activity (Fig. 2C) were significantly lower in the animals expressing IL-12, reflecting the in vivo expression data. Furthermore, no elevation in liver transaminase levels was observed (Fig. 2D). Our results indicate that the lower levels of transgene expression in the group of mice expressing IL-12 it is not due to the elimination of AAV transduced hepatocytes but rather IL-12 inhibits vector expression at a transcriptional level.

### IL-12 Expression Induces IFN-γ Production by NK and T Cells, Resulting in Downregulation of AAV-mediated Expression

**Figure 3:**
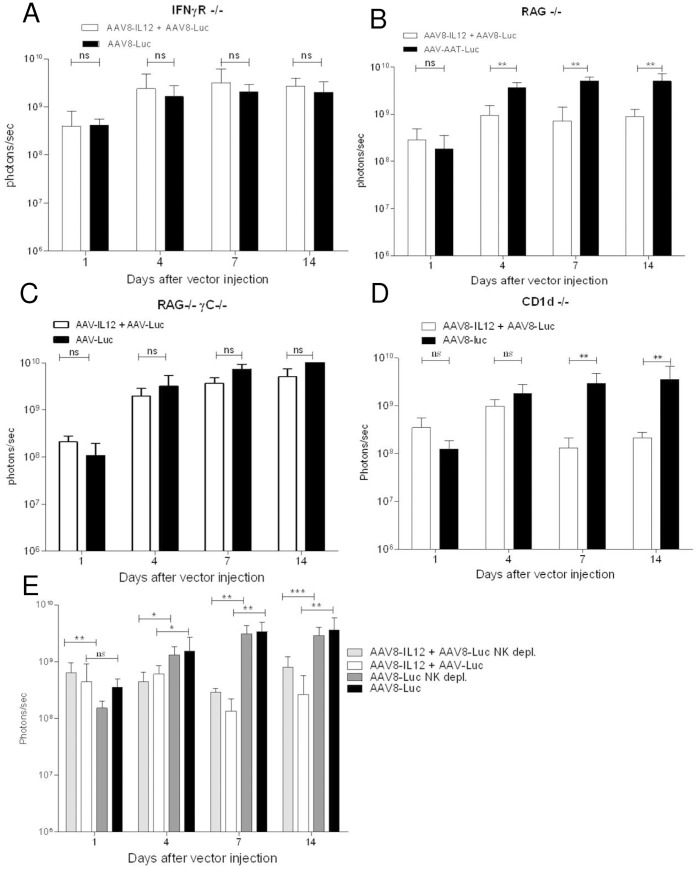
IFN-γ produced by IL12-activated NK/T cells is the main mediator of AAV-mediated transgene expression downregulation. A) IFN-**γ**R deficient mice. B) RAG deficient mice. C) RAG **γ**c deficient mice D) CD1d deficient E) C57BL/6 mice depleted or not of NK cells; received 2.5**×**1011 vg/kg of AAV8-luc vector alone or in combination with 1.5**×**109 vg/kg of AAV8-IL12 vector. As previously described, luciferase expression levels were analyzed in both groups of animals at days 1, 4, 7, and 14 using a Xenogen in vivo luminometer. Luciferase expression was quantified and represented as photons/second. The data are shown as mean ± standard deviation (SD). The differences in luciferase expression were statistically evaluated by Student t test (**p<0.01).

IFN-γ is the main mediator of many of the IL-12 biological activity. To analyze the mechanism(s) responsible for the downregulation of AAV-mediated gene expression, mice deficient in IFN-**γ** receptor α molecule were injected with AAV8-luc or AAV8-luc+AAV8-IL12. As shown in Fig. 3A, no differences were observed in luciferase expression between the two groups indicating that IFN-**γ** is one of the main mediators of the downregulation of AAV mediated gene expression. To analyze the types of cells involved in IFN-γ production RAG−/− mice (lacking T, B, and NKT cells), RAG−/− γc−/− mice (lacking T, B, NKT and NK cells), CD1d deficient mice (lacking NKT cells) were injected with AAV8-luc or AAV8-luc+AAV8-IL12 and luciferase expression was analyzed. Significant differences in luciferase expression were observed between the group no treated or treated with IL-12 when the mice were RAG−/− mice (Fig. 3B) or CD1d mice (Fig. 3D) but no when the mice were RAG−/− γc−/− (Fig. 3C), suggesting a role for NK cells. For a full elucidation of the role of NK cells, mice were depleted of NK cells using and anti-NK1.1 antibody and treated with AAV8-luc or AAV8-luc+AAV8-IL12 (Fig. 3E). The analysis of luciferase expression showed significant differences between the two groups indicating that both NK and T cells play a role in IL-12 mediated inhibition of transgene expression. Since IFN-γ is the main mediator of IL-12 inhibitory activity we next analyzed the production of IFN-γ. All the animals produced IFN-γ after vector injection except RAG-γc−/− mice, which correlates with the absence of differences in luciferase expression (Fig.S1).

### Administration of the DNA Demethylating Agent, 5-AZA, but not the Histone Deacetylase Inhibitor, TSA, Reverts the IFN-γ Inhibitory Effect

**Figure 4:**
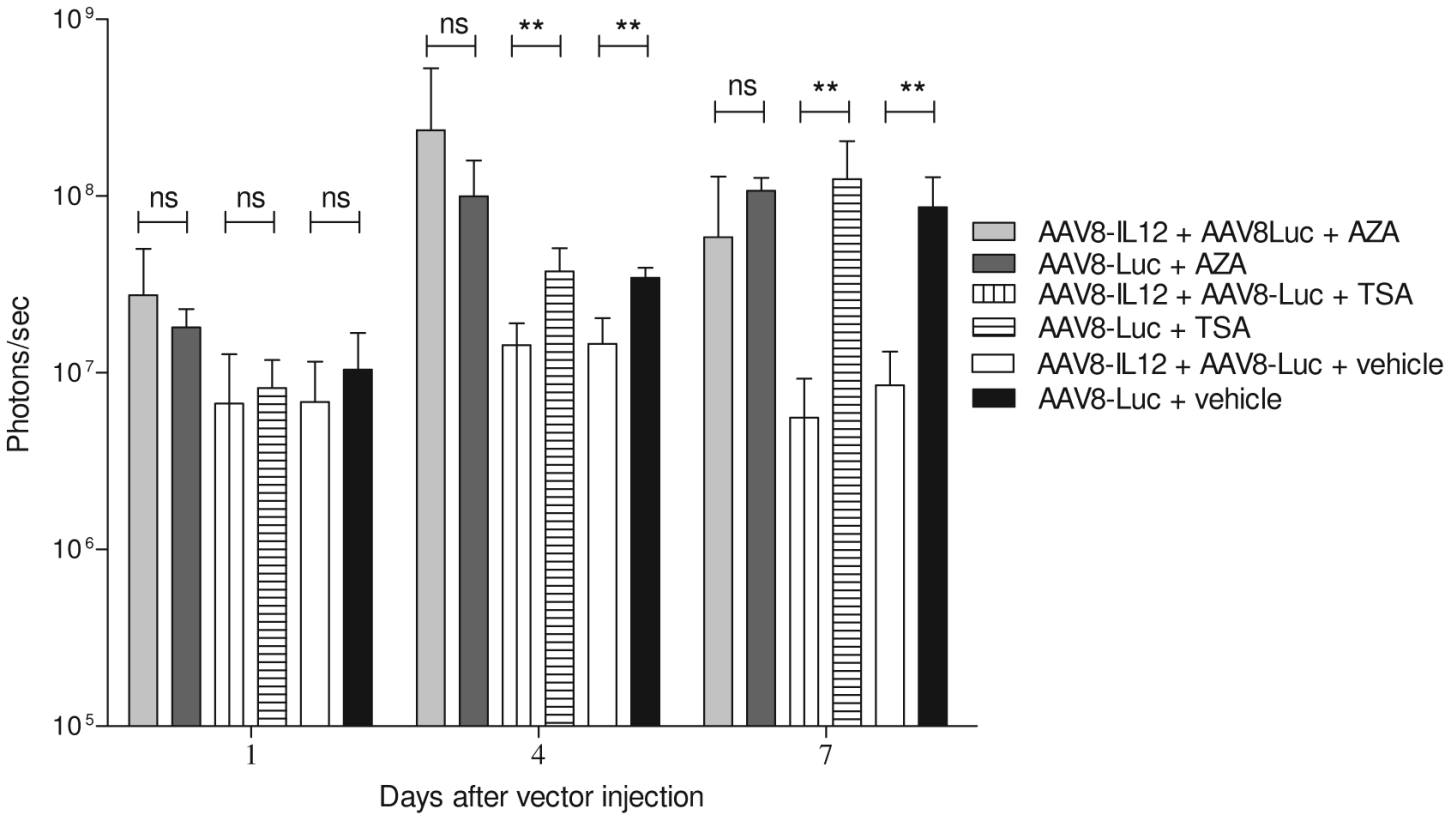
DNA demethylating agents but not histone acetylation inhibition revert the IFN-γ inhibitory effect. C57BL/6 mice received 2.5**×**1011 vg/kg of AAV8-luc vector alone or in combination with 1.5**×**109 vg/kg of AAV8-IL12 vector. One group of 5 mice treated with AAV8-luc or AAV8-luc in combination with AAV8-IL12 received 5′-Azacytidine (AZA) intraperitoneally at a dose of 1 mg/kg every 24 hours starting the day of vector injection. A second group of 5 mice treated with AAV8-luc or AAV8-luc in combination with AAV8-IL12 received 2 mg/kg of trichostatin A (TSA) were injected IP every 48 hours starting the day of vector injection. A third group of animals received only vector injection. Luciferase expression levels were analyzed in both groups of animals at days 1, 4, and 7 using a Xenogen in vivo luminometer, experiments could not be performed at longer time points due to the toxicity of 5-AZA long term treatment. Luciferase expression was quantified and represented as photons/second. The data are shown as mean ± standard deviation (SD). The differences in luciferase expression were statistically evaluated by Student t test (**p<0.01).

In previous work it has been shown that IFN-γ downregulates gene expression after hepatic administration of a plasmid or a gutless adenovirus carrying IL-12, and that a class I/II histone deacetylase inhibitor, was able to rescue liver-specific promoter activity [11]. To test the role of histone acetylation and DNA methylation in IFN-**γ** mediated AAV transgene expression downregulation, mice receiving AAV8-IL12 in combination with AAV8-luc or with AAV8-luc alone were treated with the histone deacetylase inhibitor TSA, the demethylating agent 5-AZA, or vehicle. Due to the toxicity of 5-AZA long-term treatment animals were sacrificed 7 days after vector injection. As shown in Fig. 4, no differences were observed in luciferase expression between the groups receiving IL-12 or not when the mice were treated with 5-AZA while significant differences were detected in the TSA and vehicle treated groups. These results suggest that a DNA methylation modification is affecting AAV transcriptional activity or the formation of transcriptionally active forms.

### Treatment with Anti-inflammatory Agents Reverses the Inhibitory Effect of IL-12

**Figure 5:**
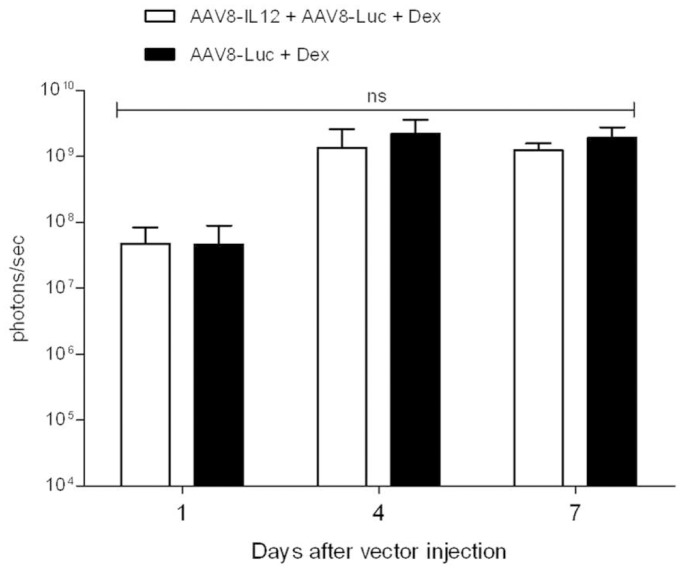
Anti-inflammatory treatment blocks the IFN-γ inhibitory effect. C57BL/6 mice received 2.5**×**1011 vg/kg of AAV8-luc vector alone or in combination with 1.5**×**109 vg/kg of AAV8-IL12 vector. Two groups of 5 mice each were injected with AAV8-luc or AAV8-luc in combination with AAV8-IL12 and received Dexamethasone (Dex) as described in methods. Luciferase expression levels were analyzed in both groups of animals at days 1, 4, and 7 using a Xenogen in vivo luminometer. Luciferase expression was quantified and represented as photons/second. The data are shown as mean ± standard deviation (SD). The differences in luciferase expression were statistically evaluated by Student t test (ns, not significant).

In order to determine if the IL-12 inhibitory effect over AAV mediated transcription can be reversed by the administration of anti-inflammatory agent; mice were treated with AAV8-IL12 in combination with AAV8-luc or with AAV8-luc alone. The anti-inflammatory reagent dexamethasone was administered before and after the administration of the vector (as described in methods). Luciferase expression was analyzed at days, 1, 4 and 7 after vector injection. No differences were observed between the two groups indicating that corticoid treatment counteracted the inhibitory effect of IL-12 induced inflammation (Fig. 5). The analysis of IFN-**γ** production in these animals showed that dexamethasone treatment significantly reduced its production (data not shown).

### IL12-induced Liver Inflammation has No Effect Over Established AAV-mediated Gene Expression

**Figure 6:**
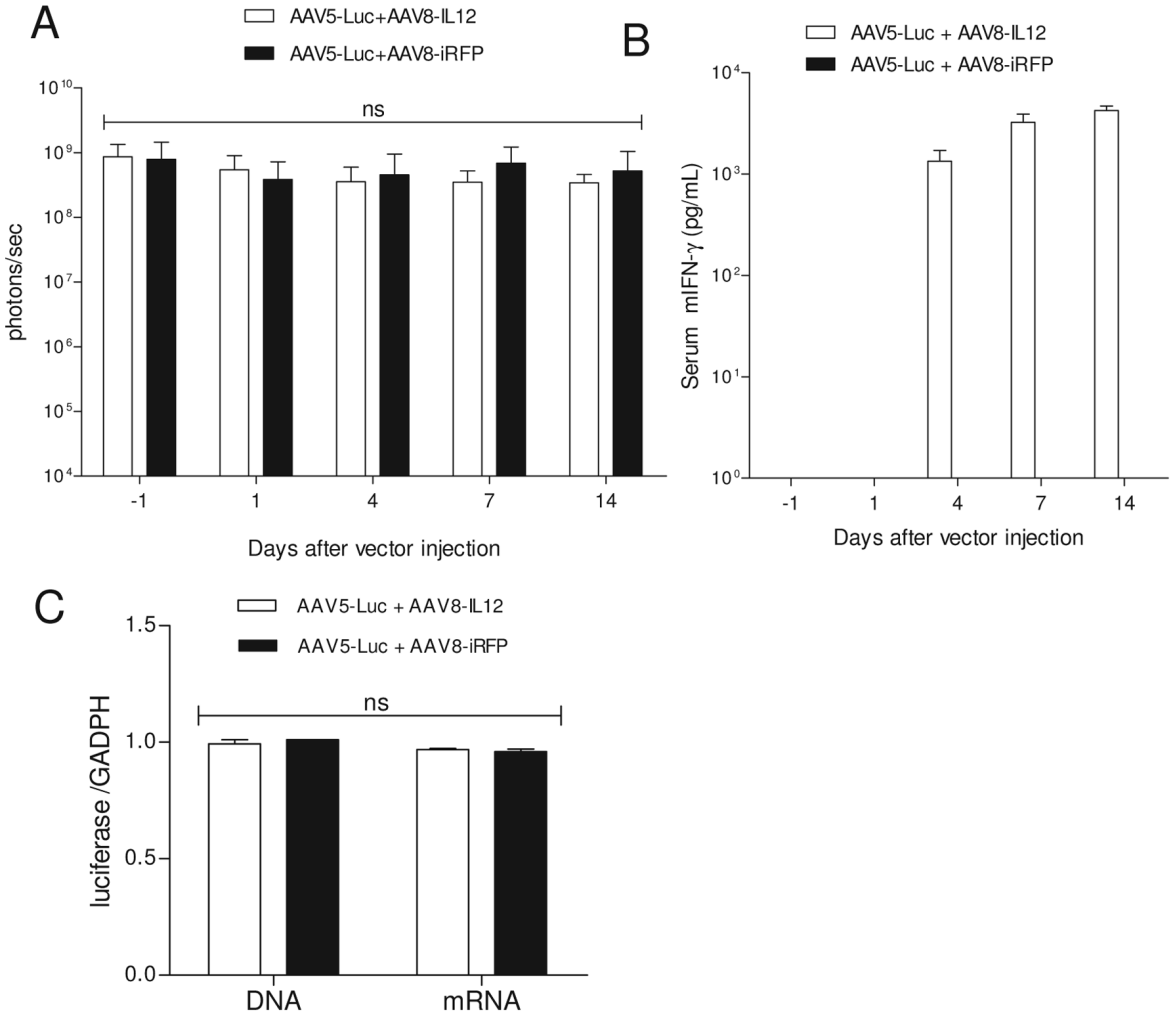
IL-12 has no effect over AAV-mediated stable expression. A) C57BL6 were intravenously injected with an AAV5-luc vector at a dose of 2.5**×**1013 vg/kg, 21 days later luciferase expression was measured (day −1) and one day (day 0) later animals (n = 5) received an intravenous injection of AAV8-IL12 or AAV8-iRFP at a dose of 1.5**×**109 vg/kg. Luciferase expression levels were analyzed in both groups of animals at days 1, 4, 7, and 14 using a Xenogen in vivo luminometer. Luciferase expression was quantified and represented as photons/second. The data are shown as mean ± standard deviation (SD). The differences in luciferase expression were statistically evaluated by Student t test (ns, not significant). B) IFN-**γ** expression levels in serum were analyzed by ELISA at days 1, 4, 7 and 14. C) Animals were sacrificed 14 days after AAV8-IL12 and AAV8-iRFP injection. Total DNA and RNA were isolated from liver and vector genome copies and luciferase messenger RNA (mRNA) copies were determined using a q-PCR specific to luciferase, normalized to GADPH copy numbers. Values corresponding to the mean copy number from five animals.

To further characterize the effect of IFN-γ induction over AAV-mediated transgene expression we administered first an AAV5 vector expressing luciferase under the control of a liver specific promoter (AAV5-Luc) at a dose of 2.5**×**10^13^ vg/kg and, when luciferase expression reached stable expression (21 days after vector injection), mice were treated with AAV8-IL12 or an AAV8 expressing the red fluorescent protein as control (AAV8-iRFP) at a dose of 1.5**×**10^9 ^vg/kg. As shown in Fig. 6A, luciferase expression was unaltered in both groups indicating that once the AAV genomes are stabilized in the hepatocyte their expression is not affected by IL-12-induced inflammation. Animals receiving AAV8-IL12 showed IFN-**γ** in serum indicating the AAV8 infection was not neutralized by anti-AAV5 antibodies (Fig. 6B). Mice were sacrificed at day 14 and viral genome and luciferase mRNA expression levels were analyzed by quantitative (qPCR) and luciferase activity was determined in liver extracts, supporting in vivo results (Fig. 6C).

## Discussion

The initial goal of this experimental work was to develop a small animal model in which AAV-mediated liver transduction is eliminated by cellular immune response against viral capsid, in an effort to reproduce the findings in the hemophilia clinical trials in which patients developed a T cell immune response against the AAV capsid protein, that resulted in the elimination of transduced hepatocytes [3] [4].

One of the major advantages of AAV vectors is their comparatively low immunogenicity profile, particularly the fact that they elicit only limited inflammatory responses, at least in experimental animal models. In fact the activation of the innate immune response due to TLR2 and TLR9 activation after recognition of AAV genome and capsid has been shown to be very modest [12]. In an attempt to activate the innate immune system and to induce the maturation antigen presenting cells (APCs) in the presence of AAV viral particles we constructed an AAV viral vector expressing IL-12, which is a potent activator of innate and adaptive immune responses. In fact, IL-12 gene transfer into tumors has been shown to induce the generation of specific CTLs and tumor rejection [9]. Here we showed that the systemic administration of very low doses of an AAV serotype 8 virus expressing IL-12 under the control of a liver specific promoter induces a strong inflammatory response in the liver, with production of inflammatory cytokines including IFN-**γ**, TNF-α or IL-1β and presence of inflammatory infiltrates (Fig. 1D). No cytokine production was detected when the reporter virus was administered alone at high doses. Furthermore, ELISpot analysis showed that IL-12 expression enhances the immune response against AAV8 capsid derived peptides. In fact the total number of IFN-**γ** producing cells after stimulation with the peptide pools covering the whole capsid protein is similar to the one induced by immunization with an adenovirus expressing AAV8 capsid proteins (Ad5-Cap8) [10]. Interestingly, the pool of peptides recognized after Ad5-Cap8 immunization or AAV8-teON-IL12 were different suggesting a different processing of the capsid. These differences might be due to the fact that when using Ad5-Cap8 the antigen is endogenously expressed, processed and presented in APCs, while after AAV administration the AAV capsid proteins must be taken up exogenously and loaded in MHC class I molecules throughout a process known as cross-presentation.

Unexpectedly, long-term transgene expression could be achieved after the administration of AAV expressing IL-12 indicating that neither the inflammatory reaction induced by IL-12, nor the immune response induced against AAV capsid proteins or the transgene, were able to eliminate AAV-transduced hepatocytes. Our results correlate with previous studies in which it was shown that, despite the presence of lytic CD8+ T cells in the liver, factor IX expression was sustained and comparable in AAV-immunized and naïve animals [12]–[15]. So far only, the adoptive transfer of AAV-cap specific CD8 T cells obtained from immunized Balb/c mice and expanded in vitro together with *in vivo* LPS stimulation after has been shown to reduce AAV-mediated transgene expression due to hepatocytes elimination [16]. Altogether these data suggest that features unique to the human immune system, compared to lower mammals may account for the difference in outcome of gene transfer observed in the clinic compared to preclinical results [17].

A comparative analysis of luciferase expression between animal receiving AAV8-luc alone in combination with AAV8-IL12 showed that no effect was seen very early after viral infection (day 1) when very little expression, if any, is coming from stabilized genomes, but later on, during the formation of transcriptionally active forms, IL-12 clearly downregulates transgene expression. In particular IFN-**γ** produced by IL-12-activated NK and T cells significantly down-regulates AAV mediated transgene expression, reaching only 10% of the expression levels of the animal injected with the reporter virus alone. In agreement with the *in vivo* data we observed a significant 10-fold reduction of messenger RNA and protein levels, however, no differences in vector genome copy numbers. These data suggest that liver inflammation has no effect on AAV8 entry into the cell or nucleus, but rather it interferes with a post-entry step. Furthermore, IL-12 expression in the liver once the AAV is transcriptionally stable has no effect over transgene expression indicating no inhibition of promoter activity. Taken together, our results suggest that IFN-**γ** interferes with AAV transcription.

Interestingly, we consistently found that the levels of luciferase expression after the administration AAV8-luc alone was approximately 10 times higher in IFN-γR and RAG deficient mice than in WT mice. It has been recently reported that AAV injection induced IL-12 production through activation TLR9, thus our results might indicate that IFN-**γ** production induced by IL-12 after AAV-mediated TLR9 activation reduced AAV-mediated transgene expression in WT animals [18]. In fact, It remains to be determined if lower transgene expression in WT animals when compared to TLR9 KO mice is due to a lower number of viral genomes or to a mechanism of transcriptional inhibition, this would provide more evidences of the possible relationship between liver inflammation and silencing of AAV-mediated transgene expression. Furthermore, we cannot discard a role for type-I IFNs, which have been frequently associated with regulation of gene expression, however, attending to the work recently published by Suzuki et al., rAAV vectors are poor inducers of type I IFNs [19].

The biological actions of IFN-**γ** are characterized by both the activation and the inhibition of gene transcription. Unfortunately, in contrast to gene activation, the mechanisms through which the cytokine suppresses gene transcription remain largely unclear. Previous studies performed by our group showed that IL-12 expression using an inducible system down-regulates the expression of the inducible promoter itself, and this effect can be partially reversed by treatment with a histone deacetylase inhibitor [11]. However, our results show that treatment with the histone deacetylase inhibitor trichostatin A has no effect, while the administration of the demethylating agent 5-aza-2′-deoxycytidine abolished IL-12-induced down-regulation of AAV-mediated transgene expression. These data suggest that IFN-γ alters the expression of a cell factor (or factors) throughout DNA methylation, which is important for the formation of transcriptionally active forms of AAV vector genomes. Additional studies are required to determine which is the mechanism induced by IFN-γ that reduced the transcriptional activity of AAV genomes.

Recently, Breous and colleagues developed a mouse model that mimics a scenario in which a subject that has received hepatic AAV-mediated gene transfer develops subsequent systemic inflammation. To do so, AAV injected animals received an adenovirus expressing the same or an irrelevant antigen in combination with several doses of TLR ligands. They found that the inflammation induced by this treatment inhibits AAV-mediated expression of transgenes in mouse liver [20]. However, their data indicate that the loss of AAV mediated transgene expression required a T cell response, most likely directed against adenoviral proteins, which in turn eliminated a significant percentage of AAV transduced hepatocytes. Similar results were previously published by Washburn and colleagues, who induced liver inflammation after AAV injection by using an adenovirus expressing LIGHT, a member of the TNF superfamily that is a potent co-stimulator of T cells [21]. Also in this study, it was unclear whether the disappearance of AAV mediated gene expression was due to a response against the adenovirus or to a direct effect over AAV genomes. Nonetheless these results are in agreement with our data, as the strong inflammatory reaction produced by TLR ligands or adenovirus injection has no direct effect over AAV mediated transgene expression [20] [21].

In conclusion, this work indicates that long-term AAV-mediated transgene expression will not be affected by the development of an inflammatory process, which may occur as a result of a viral infection or due to underlying diseases causing liver inflammation [22]. However, our work suggests that down-regulation of transgene expression following AAV mediated gene transfer could results from expression of pro-inflammatory cytokines and consequent interference with vector genome stabilization early on after gene delivery. This may not lead to loss of transduced hepatocytes but may result in lower levels of transgene expression. For this reason, before AAV administration, it will be crucial to assess the baseline levels of inflammation in the target tissue, in this case the liver. This is important particularly because many disorders under consideration for hepatic gene transfer, such as chronic hepatitis infection or hepatic genetic disorders, like α1-antitripsin deficiency, present with some levels of underlying liver disease, which may reduce efficiency of liver-derived transgene expression. In these cases the use of an anti-inflammatory treatment should be carefully evaluated in order to increase vector transduction efficiency.

## Materials and Methods

### Animals and Animal Manipulation

Experiments were performed with 6–8 weeks-old female C57BL/6 purchased from Harlan Laboratories (Barcelona, Spain), knockout mice for the α chain of the IFN-γ receptor (IFNγR KO mice), RAG-1-deficient (RAG1−/−) mice, CD1d-deficient (CD1d−/−) mice and RAG−/− γc−/− mice. Mice were bred and maintained under pathogen-free conditions in the animal facility of the University of Navarra. The experimental design was approved by the Ethical Committee for Animal Testing of the University of Navarra. Mice were injected intravenously with the AAV viruses. For all procedures, animals were anesthetized by intraperitoneal (IP) injection of a mixture of xylacine (Rompun 2%, Bayer) and ketamin (Imalgene 50, Merial) 1∶9 v/v. Blood collection was performed by bleeding in the retro-orbital plexus, and serum samples were obtained by supernatant recovery after centrifugation of total blood. Doxicycline treatment for the induction of the AAV8-TetON-IL-12 vector, was performed by a first IP injection of 50 mg/kg of doxycycline (Sigma) followed by oral administration of a mixture of 2 mg/ml of doxycycline with a 5% of sucrose for 6 days. Treatment with 5′-Azacytidine (Sigma) was administered by IP injection of 1 mg/Kg every 24 hours. For the inhibition of histone deacetylation, 2 mg/kg of trichostatin A (Alomon Laboratories) were injected IP every 48 hours. Dexamethasone was administered IP at a dose of 10 mg/kg one day before and the same day of vector injection, at a dose of 5 mg/kg 1 and 2 days after vector injection, and at a dose of 1 mg/kg the following days.

Animals were euthanized by cervical dislocation after being anesthetized at the indicated time points. Liver samples were collected for histological analysis and for nucleic acid and protein extraction.

### Viral Constructs and Vector Production and Purification

Recombinant AAV vectors were constructed with a transgene cassette encoding the IL-12 single chain [9] or the reporter gene luciferase under the regulation of a chimeric liver specific promoter composed of the human α1-antitrypsin promoter (AAT) with regulatory sequences from the albumin enhancer (Ealb) [23]. The transgene cassette was flanked by AAV2 wild type inverted terminal repeats. rAAV8 vectors with wild-type AAV2 ITRs were produced by polyethylenimine (PEI) mediated cotransfection in HEK-293 cells. For each production a mixture of plasmids, 20 µg of pro-AAV plasmid and 55 µg pDP8.ape (PlasmidFactory GmbH & Co. KG, Bielefeld Germany), was transfected into 293 T cells 15 cm plate using linear PEI 25 kDa (Polysciences, Warrington, PA, USA) as described [9]. The cells were harvested 72 hr after transfection and virus was released from the cells by three rounds of freeze–thawing. Crude lysate from all batches was then treated with Benzonase (50U/ml crude lysate) for 1 hr at 37°C and then kept at −80°C until purification. Purification of crude lysate was performed by iodixanol gradients according to the method of Zolotukhin and colleagues [24]. The purified batches were concentrated and diafiltrated by cross-flow filtration (Spectrum Laboratories, Rancho Dominguez, CA) with a molecular mass cut-off of 400 kDa. The batches were then concentrated further by passage through Centricon tubes (YM-100; Millipore, Bedford, MA) to a final concentration of 1**×**10^12^ vg/ml, as determined by quantitative polymerase chain reaction (q-PCR). After concentration, the viral batches were filtered (pore size, 0.22 mm) and stored at −80°C. Viral titers in terms of genome copies per milliliter were determined by Q-PCR, performed three times in triplicate at three different dilutions.

The adenovirus expressing AAV8 capsid proteins was produced as described [10].

### Bioluminescence Imaging

Mice were immobilised with IP anesthesia (a mixture of xylacine and ketamine). The substrate D-luciferin (150 µg/kg dissolved in phosphate-buffered saline; Promega, Madison, USA) was injected IP. Ten minutes later, animals were placed in the dark chamber for light acquisition in an IVIS CCD camera system (Xenogen) and analysed with the Living Image 2.20 software package (Xenogen). A region of interest (ROI) covering the whole animal was defined, and quantification of light emission was performed in photons/second. The same ROI was used for all the animals in the different experiments. Time exposure ranged from 1 second to 5 minutes depending on light intensity.

### Luciferase Measurement

Organ sections were frozen in liquid nitrogen until processed. Tissue was homogenised in Luciferase Lysis Reagent (Promega). Samples were centrifuged 15 sec at 12000g. Supernatant was collected and measured in a tube luminometer (Lumat LB 9507, from EG&G Bathold). Total proteins were quantified using the Bradford assay using bovine serum albumin as a standard (Thermo Scientific).

### NK Depletion

Mice were NK-depleted by IP administration of 500 µg of the anti-mouse NK1.1 antibody (PK136, BioXcell) 2 days before virus injection and every 48 hours. Depletion levels of circulating NK cells were determined to be >98% by flow cytometry of whole blood.

### Determination of IL-12 and IFN-γ

Serum concentrations of murine IL-12 and IFN-γ were determined using OptEIA Mouse IL-12 (p70) and Mouse IFN-γ ELISA Kits (BD Bioscience-Pharmigen, San Diego, CA) according to manufacturer's instructions.

### Determination of Serum Levels of AST and ALT

Alanine aminotransferase (ALT) and aspartate aminotransferase (AST) serum levels were analyzed in a Hitachi Automatic Analyzer (Boehringer Mannheim, Indianapolis, IN).

### Total DNA and RNA Extraction

To quantify the number of viral genomes, total DNA (tDNA) was extracted from frozen livers using the QIAamp DNA Mini Kit (Qiagen) following the manufacturer's instructions. To analyze transgene expression, total RNA was isolated from frozen livers using the TRIzol Reagent (Invitrogen) according to manufacturer's instructions. Both nucleic acids were quantified after extraction.

### Viral Genome and Transgene Expression Quantification

Extracted RNA was pre-treated with DNAse I (Invitrogen) and retro-transcribed into complementary DNA (cDNA) using M-MLV reverse-transcriptase (Invitrogen). Copies of luciferase in tDNA and cDNA were analyzed by quantitative PCR (qPCR) using iQ SYBR Green Supermix (BioRad) in a CFX96 Real-Time Detection System (BioRad). All data were normalized to GAPDH (Glyceraldehyde-3-phosphate deshidrogenase) and primers used were: GAPDH 3′- TGCACCACCAACTGCTTA, GAPDH 5′- GGATGCAGGGATGATGTTC, Luciferase 3′- TCTGAGGAGCCTTCAGGATT, Luciferase 5′- TTTTGGCGAAGAAGGAGAAT.

### Histology and Immunohistochemistry

Liver sections were fixed in 4% paraformaldehyde (Panreac), embedded in paraffin, sectioned (3 µm), and stained with hematoxylin and eosin. Sections were mounted and observed by light microscopy.

### Quantification of Specific IFN-**γ** Producing Cells by ELISpot Assay

Splenocytes were isolated as previously described [9]. Briefly, spleens were mechanically disrupted and, after centrifugation, red blood cells were lysed using ACK lysing buffer. Cells obtained were resuspended in complete medium (RPMI-1640 supplemented with 10% of fetal bovine serum, 1% of Penicillin/Streptomycin and L-Glutamine). The AAV8 VP1 capsid peptide library was generated as 15-mers overlapping by 12 amino acids (Mimotopes); we separated the AAV-8 peptide library into 15 consecutive pools each containing 10 peptides for the analysis, except pool number 15 that contains 6 peptides. Splenocytes (5**×**10^5^ cells/per well) obtained were seeded in each ELISpot well in complete medium and stimulated for 24 h at 37°C and 5% CO2 with pools of peptides (2 µg/ml each), with a previously described H2b epitope from AAV8 capsid protein NSLANPGIA (5 µg/ml) (Sabatino), with PMA plus ionomycin (0.05 µg/ml and 1 µg/ml, respectively) for positive controls or in absence of peptides for negative controls. The interferon (IFN)-γ ELISpot assay was performed according to the manufacturer's instructions (BD Biosciences, San Jose, CA). The number of spots corresponding to IFN-**γ** secreting cells was determined using an ELISpot automatic reader (Immunospot CTL, Germany). Samples were scored positive if numbers of spots/well in presence of the peptide pools were >3 times higher than number of spots/well that were cultured without peptides and if numbers of spots in experimental wells were at least 3 SD above those in control wells.

### Statistical Analysis

The data are presented as mean values ± standard deviation and all data were analyzed for significance by the Student t test (p<0.05 was considered significant) with the GraphPad Prism 5.0 software.

## Supporting Information

Figure S1 **IFN-γ expression levels in serum were analyzed by ELISA in C57BL/6 WT mice, RAG deficient mice, RAG γc deficient mice and CD1d deficient 1, 4, 7 and 14 days after injection of 2.5×1011 vg/kg of AAV8-luc vector alone or in combination with 1.5×109 vg/kg of AAV8-IL12 vector.**
(TIF)
